# Investigation of Timing to Switch Control Mode in Powered Knee Prostheses during Task Transitions

**DOI:** 10.1371/journal.pone.0133965

**Published:** 2015-07-21

**Authors:** Fan Zhang, Ming Liu, He Huang

**Affiliations:** Joint Department of Biomedical Engineering, North Carolina State University and University of North Carolina at Chapel Hill, Raleigh, North Carolina, United States of America; West Virginia University, UNITED STATES

## Abstract

Current powered prosthetic legs require switching control modes according to the task the user is performing (e.g. level-ground walking, stair climbing, walking on slopes, etc.). To allow prosthesis users safely and seamlessly transition between tasks, it is critical to determine when to switch the prosthesis control mode during task transitions. Our previous study defined critical timings for different types of task transitions in ambulation; however, it is unknown whether it is the unique timing that allows safe and seamless transitions. The goals of this study were to (1) systematically investigate the effects of mode switch timing on the prosthesis user’s performance in task transitions, and (2) identify appropriate timing to switch the prosthesis control mode so that the users can seamlessly transition between different locomotion tasks. Five able-bodied (AB) and two transfemoral (TF) amputee subjects were tested as they wore a powered knee prosthesis. The prosthesis control mode was switched manually at various times while the subjects performed different types of task transitions. The subjects’ task transition performances were evaluated by their walking balance and success in performing seamless task transitions. The results demonstrated that there existed a time window within which switching the prosthesis control mode neither interrupted the subjects’ task transitions nor disturbed their walking balance. Therefore, the results suggested the control mode switching of a lower limb prosthesis can be triggered within an appropriate time window instead of a specific timing or an individual phase. In addition, a generalized criterion to determine the appropriate mode switch timing was proposed. The outcomes of this study could provide important guidance for future designs of neurally controlled powered knee prostheses that are safe and reliable to use.

## Introduction

The advent of powered prosthetic legs has demonstrated great promise to significantly improve the mobility of people with lower limb amputations [1–5]. With powered devices, lower limb amputees are now capable of performing a variety of locomotion tasks more easily and efficiently, such as staircase climbing and slope walking, which are difficult or even impossible to perform with a passive prosthesis. This is mainly because powered prosthetic legs can generate positive net power over gait cycles during ambulation, a feature that is absent in traditional passive devices.

Current commercialized powered lower limb prostheses employ intrinsic control, i.e. control based on intrinsic mechanical feedback [1–2]. Such control is mode-based. Each control mode corresponds to one type of locomotion task (e.g. level-ground walking, stair climbing, walking on slopes, etc.). To allow prosthesis users to transition from one task to another, prosthesis modes need to be switched accordingly. Traditional manual mode switching, such as using a remote key fob [6] or performing extra body motions [1], is functionally viable; however, the manual approaches are cumbersome and sometimes unreliable. To make the prostheses easy to use, a hierarchical prosthesis controller has been proposed and adopted [5, 7, 8]. The hierarchical control structure mainly consists of (1) a high-level controller in which an intent recognition interface is designed to recognize the user’s intended locomotion tasks, and (2) a low-level intrinsic controller that generates the appropriate joint motions according to the user’s task. In the design of high-level controllers, significant efforts have been focused on improving the accuracy in identifying the user’s locomotion tasks and predicting the user’s task transitions [5, 7, 9–13]. Various approaches based on EMG signals from residual muscles [3, 9, 14, 15], intrinsic mechanical measurements [7], and neuromuscular-mechanical fusions [5, 10, 12] have been explored.

In the design of hierarchical prosthesis control, determining the correct timing to switch prosthesis control modes during the user’s task transitions is critical because incorrect mode switch timing might interrupt the user’s task performance and threaten the user’s stability and safety. Varol *et al*. [7] reported a method to identify user intent to switch the control mode for walking, standing, and sitting. In that study, the prosthesis was allowed to switch the control mode in a certain phase for each type of task transition. The entire hierarchical control was evaluated on one transfemoral amputee. All the tested task transitions were successfully performed. However, the included tasks, such as standing and sitting, were quasi-static, compared to ambulation. It is unknown what mode switch timing in prosthesis control is appropriate in order to ensure the user’s safety in transitions among ambulation tasks (e.g. level-ground walking and slope walking). In our previous work [10] based on human biomechanics, we defined the critical timings for different types of transitions among tasks, such as level-ground walking, ramp ascent/descent, stair ascent/descent, and stepping over an obstacle. For transitions from the level-ground walking task, the critical timing was defined at the beginning of swing phase so that the knee joint could generate the proper flexion torque and prevent tripping over the obstacle, staircase, or incline. For all transitions to the level-ground walking task, the critical timing was chosen at initial contact of the prosthetic foot on level ground because the prosthetic side of the leg was still ascending/descending a staircase or ramp before this gait event. Our defined critical timing was applied and validated in recent studies [5, 16]. With the defined critical timings for control mode switching, transfemoral amputees were able to seamlessly perform the task transitions when ambulating on level ground, stairs, and ramps.

Our previous study [10] presumed that prosthesis control modes have to be switched at a specific critical timing so that prosthesis users can seamlessly and safely perform task transitions in ambulation. Is the defined critical timing to enable safe and seamless task transitions unique? What if the prosthesis switches the control mode earlier or later than the critical timing? To the best of our knowledge, few studies have tried to answer these questions by systematical study of control mode switch timing for powered lower limb prostheses.

Hence, in this study we aimed to (1) systematically investigate the effects of mode switch timing on prosthesis control and the user’s performance in task transitions, and (2) identify appropriate prosthesis control mode switch timings that enable the users to seamlessly adapt to the changing terrains while walking. A locomotion mode simulator was designed to manually switch prosthesis control modes at different timings during task transitions. The effects of different mode switch timings on the user’s performance in task transitions and the prosthesis were quantified based on data collected from five able-bodied (AB) subjects and two patients with unilateral transfemoral (TF) amputations. The results of this study could provide important guidance for controlling powered lower limb prostheses, and further enhance the prosthesis user’s safety and confidence when operating powered prosthetic legs.

## Methods

### A. Design and Control of a Powered Knee Prosthesis

A prototypical powered knee prosthesis with a passive ankle joint [17] was used as a test bed in this study. The knee joint was constructed from a moment arm supported by an actuator and an aluminum pylon. A direct-current (DC) motor was used to drive the knee joint motion (i.e. flexion and extension) through a ball screw. Intrinsic sensors, including a potentiometer, an encoder, and a 6 degree-of-freedom (DOF) load cell, were instrumented on the prosthesis for intrinsic prosthesis control. The prosthesis was tethered to and controlled by LabVIEW (National Instruments, TX) running on a PC. More details about the prosthesis design can be found in [17].

**Fig 1:**
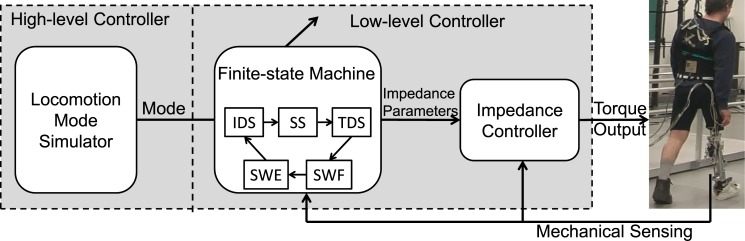
The prosthesis controller with a locomotion mode simulator.

The control structure of the powered prosthesis contained two hierarchies as demonstrated in Fig 1. The function of the high-level controller was to recognize the prosthesis user’s intended tasks and determine the control mode in the low-level intrinsic controller accordingly. In the low-level controller, a finite-state machine (FSM) and impedance control were designed to modulate the knee joint impedances based on the user’s task (output of high-level control) and current state (gait phase). The control goal was to ensure that the prosthetic knee acted as a passive spring-damper-system with predefined impedance (i.e. stiffness, damping coefficients, and equilibrium position) that approximated the biological knee impedance during ambulation [17, 18]. Five states were defined, each of which corresponded to one of the five defined gait phases: initial double support (IDS), single support (SS), terminal double support (TDS), swing flexion (SWF), and swing extension (SWE). The ground reaction force, measured by the load cell, and knee kinematics (i.e. knee angle and angular velocity) were used to trigger the state transitions. Given a control mode and current state, predefined impedance parameters were sent to the impedance controller to generate the desired torque to drive the prosthetic knee. The applied impedance control in this study was the same as that reported in previous studies [7, 18].

To systematically investigate the effects of control mode switch timings, a locomotion mode simulator was designed to simulate the function of the high-level controller and generate different prosthesis control mode switch timings.

### B. Investigated Task Transitions and Mode Switch Timing

Walking on level-ground and negotiating inclines are frequently encountered locomotion tasks in lower limb amputees’ daily lives. In this study, four types of task transitions were investigated: transitions from level-ground walking to ramp ascent and descent (W→RA and W→RD), and transitions from ramp ascent and descent to the task of level-ground walking (RA→W and RD→W).

**Fig 2:**
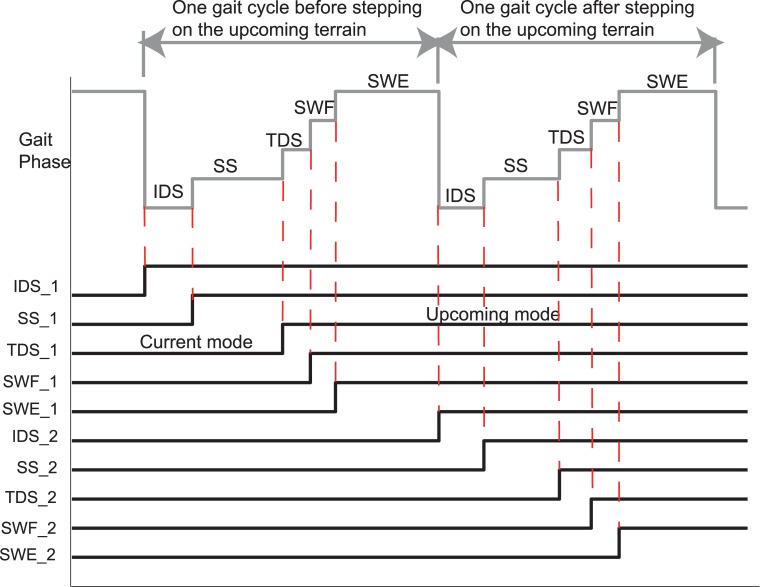
Illustration of investigated mode switch timings across two gait cycles.

To investigate the effects of control mode switch timing during task transitions, the time duration between one full gait cycle before and after the prosthetic foot stepped on the upcoming terrain was studied. Herein, one gait cycle was defined as the time duration from foot contact to the next foot contact. The locomotion mode simulator triggered the prosthesis control mode change at the beginning of a randomly selected gait phase within these two gait cycles. As a result, 10 transition timings (i.e. IDS_1, SS_1, TDS_1, SWF_1, SWE_1, IDS_2, SS_2, TDS_2, SWF_2, and SWE_2) were investigated as shown in Fig 2.

### C. Participants and Measurements

**Fig 3:**
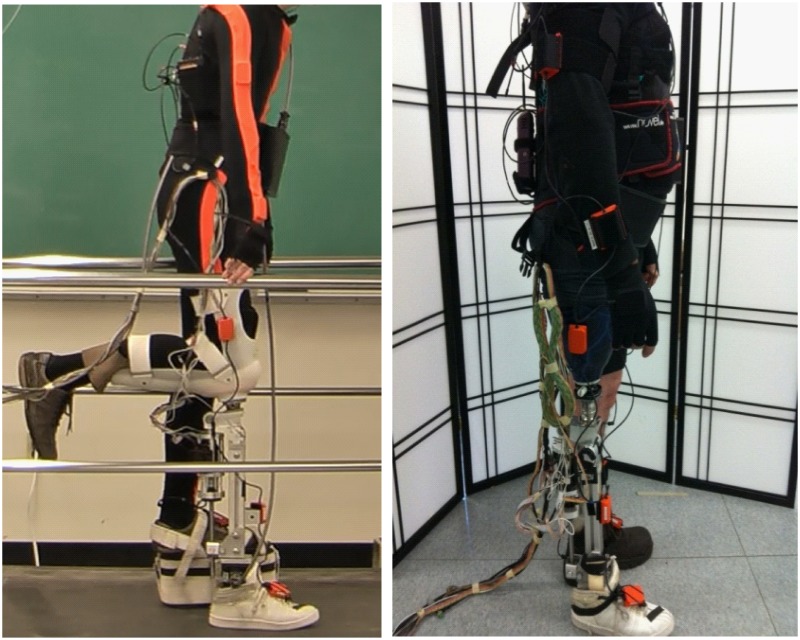
Experimental Setup on one able-bodied subject AB 01(left) and one TF amputee TF01(right).

This study was approved by the Institutional Review Board (IRB) and conducted with the written informed consent of all subjects. Five able-bodied subjects (AB01-05) and two patients with unilateral transfemoral amputations (TF01-02) were recruited. The recruited AB subjects were all healthy males free from orthopedic or neurological pathologies. The average age was 28.6 (±5.3) years; the average height was 181.6 (±4.8) cm; the average weight was 82.3 (±11.7) kg. TF01 (age: 60 years; height: 175.3 cm; weight: 75.8 kg) was a male amputee who was 33 years post-amputation; TF02 (age: 41 years; height: 162.2 cm; weight: 65.7 kg) was a female amputee who was 32 years post-amputation. They both used passive prostheses in daily life. In the experiments, the TF subjects wore suction prosthetic sockets, which were attached to the powered prosthetic leg. A special customized L-shaped bent-knee adaptor was designed for AB subjects so that they could walk with the powered prosthesis. The experimental setup is shown in Fig 3.

In the experiment, the mechanical sensor measurements from the prosthesis were recorded for intrinsic control and for evaluation purposes. An inertial measurement unit (IMU)-based 3D motion capture system (Xsens Technologies B.V., Enschede, Netherlands) was employed to capture the full-body kinematics of the subjects. The kinematic measurements were used to evaluate the user’s walking balance during task transitions (see Section II. E). All the measurements were sampled at 100Hz and synchronized. The experimental sessions were also video-taped.

### D. Experimental Protocol

Each subject was trained for at least 10 hours to walk with the prototypical powered prosthesis prior to the experiment. The training was conducted by a physical therapist and the experimenters. This training was necessary because (1) AB subjects were not regular prosthesis users and they needed to learn how to walk with a prosthesis; and (2) amputees had to re-adapt to the powered device since they were accustomed to using passive devices. In the training procedure, the intrinsic controller impedance parameters for each control mode and each state (gait phase) were obtained for each individual subject. All the subjects were able to adapt to the powered prosthesis and generate a consistent gait pattern before the experiment.

The experiment was conducted in a laboratory environment. A total of 120 trials were conducted during one experimental session for each subject. In each trial, the subject was asked to first walk on one terrain and then transition to another. The prosthesis control mode was switched by the locomotion mode simulator at a randomly selected timing during the transition period (indicated in Fig 2). The same type of task transition with the same mode switch timing was tested three times. For level-ground walking, the subject was asked to walk on a straight pathway; for ramp ascent/descent, the subject walked on a 10-foot ramp with an 8-degree inclination angle. To protect the subjects, a fall-arrest harness was applied. In addition, rest periods were allowed between trials so that subjects would not become fatigued.

### E. Effects of Control Mode Switch Timing on Prosthesis Users

To identify the effects of control mode switch timing on the prosthesis users, each subject’s gait performance during task transitions was evaluated by the subject’s walking balance and success in performing seamless task transitions. If the subject could continuously complete a task transition without stopping during the transition period, the transition was considered to be successful. Each subject’s walking balance was evaluated by obtaining subjective feedback and calculating the full-body angular momentum as an objective balance quantification index. Each subject was asked to verbally report his/her feeling about walking balance right after the task transition was completed in each trial. The subjective feedback (either stable or unstable) was recorded accordingly.

The full-body angular momentum was calculated to objectively evaluate the subject’s walking balance. This quantification index has been used for the analysis of human walking balance [19, 20]. The full-body kinematic measurements monitored by a 3D motion capture system (see Section IIB) were used to calculate the angular momentum. A simplified human model was built in this study. This model was composed of 12 rigid body segments, including the head, trunk, and bilateral upper arms, forearms, thighs, shanks, and feet. Anthropometry was measured from each subject to reconstruct the representative model. The measurements included body weight, height, head circumference, and segment lengths and circumference of arms, forearms, trunk, thighs, and shanks. The mass of each segment was estimated by using the modified Hanavan model described in [21]. The full-body's angular momentum was calculated as the sum of each individual segment's angular momentum about the full-body’s center of mass (COM). The position of the full-body’s COM was calculated as a sum of the products of each individual segment's relative masses and COM locations [20]. Details about the calculation can be found in [20]. The full-body angular momentum in the sagittal plane (“+”: posterior and “-”: anterior) was used to quantify a subject’s walking balance. The subject was reported as unstable if the observed angular momentum exceeded a defined normal range. In this study, the defined normal range for each subject was between the maximum angular momentum in both the anterior and posterior directions as measured in a trial in which the subject performed task transitions and the prosthesis control mode was switched at the critical timing as defined in the previous study [10].

### F. Effects of Mode Switch Timing on Prosthesis

Mechanical work is an important biomechanical property and has been widely used in human gait analysis [22–24]. In our previous work [8], large mechanical work changes at the knee joint when the prosthesis incorrectly switched modes could disrupt the prosthesis user’s walking balance in steady states (i.e. states in which the subject continuously performed one task). In the present study, we expect the same mechanical metric was also applicable to evaluating the effects of mode switch timing of the prosthesis during task transitions. The mechanical work at the knee joint was calculated as the time integration of the knee joint torque multiplied by the joint angular velocity over a certain period [22]. The mechanical work change was defined as the difference of mechanical work when the prosthesis switched modes at the timing that caused gait instability and the one that allowed the prosthesis users to safely and seamlessly perform task transitions. In this study, the temporal difference between the unstable timing and stable timing was one or multiple early triggered or delayed gait phases. For example as illustrated in Fig 2, compared to the timing TDS_1, the timing IDS_1 was two phases (IDS and SS) earlier. Therefore, only the mechanical work changes over these shifted phases were calculated and analyzed. In addition, the mechanical work change value was normalized by each subject’s body weight.

## Results

In total, 120 task transitions were tested for each AB or TF subject in this study. For all the studied mode switch timings, each subject was able to successfully complete the task transitions without stopping, although some of the timings were observed to disturb the subject’s gait stability.

**Fig 4:**
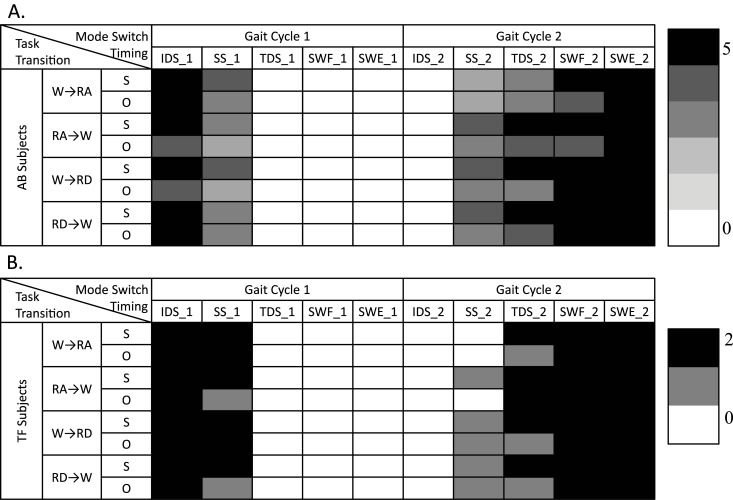
The Number of (A) AB and (B) TF Subjects whose Subjective Feedback (S) and Quantified Balance Index (O) Reported Gait Instability when the Prosthesis Mode Switched at Different Timings. White area indicates none of the subjects showed gait instability, evaluated either subjectively (S) or objectively (O).

Fig 4 showed the effects of mode switch timing on the subjects’ gait stability in task transitions. Each subject performed 3 trials for each combination of task transition type and mode switch timing. Fig 4 listed the number of AB and TF subjects, who demonstrated gait instability in at least one out of 3 trials based on the subjective feedback (S) and objective balance measurements (O). White area (denotes ‘0’) denoted that none of the AB or TF subjects reported gait instability. Clearly, for each individual type of task transition there was a time window (about 3–4 gait phases) within which switching the control mode permitted smooth and safe task transitions in all the test subjects. It is noteworthy that the previously defined critical timing [10] was within this safe window for switching modes. For example, for W→RA/RD, the critical timing was defined as the beginning of swing phase (i.e. SWF_1 in Fig 4), which was within the safe window; for RA/RD→W, the critical timing, defined as the initial ground contact of the prosthetic foot (i.e. IDS_2 in Fig 4), also fell within the safe window for mode switching.

As shown in Fig 4, we also demonstrated that subjective detection of walking instability was more sensitive than detection based on a quantitative balance index. All mode switches that perturbed the subject’s full-body angular momentum out of the defined normal range were also reported as unstable transitions based on the subject’s feedback. Nevertheless, not all transitions that were subjectively reported as unstable caused the full-body angular momentum to exceed the defined normal range.

For AB subjects, the time windows that were observed to allow safe and smooth transitions between tasks included TDS_1, SWF_1, SWE_1, and IDS_2, as shown in Fig 4. The identified safe time windows for mode switches, derived from TF subjects, were consistent with those observed from AB subjects in general. A slight difference was observed for task transitions W→RA and RA→W.

Tables 1 and 2 showed the amount of mechanical work change for the mode switch timings that were reported to cause gait instability, compared to ones that allowed safe and seamless task transitions. The results from a representative transition type (W→RA) were demonstrated in these tables. Only timings reported to cause gait instability (according to the results in Fig 4) were demonstrated. If timings were earlier than the safe time window identified in Fig 4, they were compared to the left boundary (i.e. TDS_1) of the safe time window to calculate the mechanical work change; if the timings were after the identified time window, they were compared to the right boundary (i.e. IDS_2) of the safe time window to calculate the mechanical work change. For example, for task transition W→RA, the timing IDS_1, reported to disturb the subject’s gait stability, was compared to TDS_1 (initial phase of the safe time window) when calculating the mechanical work change. According to the timing illustration in Fig 2, IDS_1 switched the prosthesis control mode two gait phases (IDS and SS) earlier than TDS_1. Therefore, only the mechanical work changes in these two early triggered phases were quantified and demonstrated in Tables 1 and 2; whereas, the mechanical work changes in other phases were not calculated and indicated as “-”. For each timing that reportedly caused gait instability (each row in Tables 1 and 2), there was at least one gait phase in which the mechanical work change was out of the tolerable range identified in our previous work [8] (highlighted by gray area). This was also observed for all other types of the tested task transitions. Our previous study already found that large changes in the mechanical work performed by the prosthesis knee joint could obviously disturb the prosthesis user’s walking stability during steady ambulation. Herein, we concluded that the disturbance on walking stability during task transitions was also related to the large mechanical work change generated by inappropriate mode switch timing.

**Table 1:** Change of Mechanical Work on AB Subjects in Each Gait Phase when the Prosthesis Control Mode Was Switched from Level-ground Walking to Ramp Ascent (W→RA) at Timings that Were Reported Unstable (IDS_1, SS_1, SS_2, TDS_2, SWF_2, and SWE_2).

	One Gait Cycle before Transition					One Gait Cycle after Transition				
Unstable Task Transitions	IDS	SS	TDS	SWF	SWE	IDS	SS	TDS	SWF	SWE
**IDS_1**	**0.072** *	**0.019** *	**-**	**-**	**-**	**-**	**-**	**-**	**-**	**-**
**SS_1**	**-**	**0.015** *	**-**	**-**	**-**	**-**	**-**	**-**	**-**	**-**
**SS_2**	**-**	**-**	**-**	**-**	**-**	**-0.059** *	**-**	**-**	**-**	**-**
**TDS_2**	**-**	**-**	**-**	**-**	**-**	**-0.059** *	**-0.007** *	**-**	**-**	**-**
**SWF_2**	**-**	**-**	**-**	**-**	**-**	**-0.059** *	**-0.007** *	**-0.060**	**-**	**-**
**SWE_2**	**-**	**-**	**-**	**-**	**-**	**-0.059** *	**-0.006** *	**-0.059**	**-0.094**	**-**

Note: The values were averaged across five AB subjects.

“*” indicated that the change of mechanical work was out of the tolerable range that was observed in the previous study.

“-” meant that the mechanical work change was not calculated. The unit of mechanical work change was J/Kg.

**Table 2:** Change of Mechanical Work on TF Subjects in Each Gait Phase when the Prosthesis Control Mode Was Switched from Level-ground Walking to Ramp Ascent (W→RA) at Timings that Were Reported Unstable (IDS_1, SS_1, TDS_2, SWF_2, and SWE_2).

	One Gait Cycle before Transition					One Gait Cycle after Transition				
Unstable Task Transitions	IDS	SS	TDS	SWF	SWE	IDS	SS	TDS	SWF	SWE
**IDS_1**	**0.084** *	**0.017** *	**-**	**-**	**-**	**-**	**-**	**-**	**-**	**-**
**SS_1**	**-**	**0.014** *	**-**	**-**	**-**	**-**	**-**	**-**	**-**	**-**
**TDS_2**	**-**	**-**	**-**	**-**	**-**	**-**	**-0.010** *	**-**	**-**	**-**
**SWF_2**	**-**	**-**	**-**	**-**	**-**	**-**	**-0.010** *	**-0.049**	**-**	**-**
**SWE_2**	**-**	**-**	**-**	**-**	**-**	**-**	**-0.009** *	**-0.049**	**-0.122**	**-**

Note: The values were averaged across two TF subjects.

“*” indicated that the change of mechanical work was out of the tolerable range that was observed in the previous study.

“-” meant that the mechanical work change was not calculated. The unit of mechanical work change was J/Kg.

**Fig 5:**
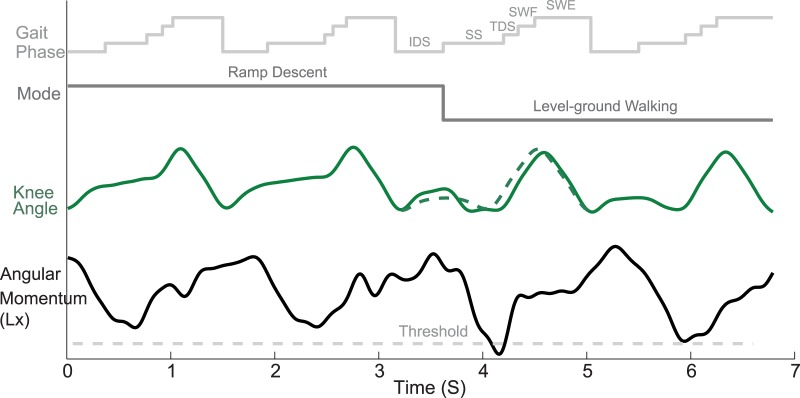
One representative trial from one TF amputee subject (TF01) when the prosthesis mode was switched from ramp descent to level-ground walking at the beginning of single stance phase after he stepped on the level ground (SS_2 indicated in Fig 2).

Fig 5 illustrated one representative trial from one TF subject (TF01) for whom, while transitioning from ramp descent to level-ground walking, the prosthesis was triggered to switch modes at SS_2 (as shown in Fig 2). After the prosthetic leg stepped on the level-ground, the prosthesis knee joint generated excessive stance flexion motion compared to the knee motion during the seamless and safe task transition (indicated by the green dashed line curve in Fig 5). Around 400ms after the prosthesis switched modes, the subject’s full-body angular momentum in the sagittal plane demonstrated an obvious change and exceeded the defined normal range (threshold). This subject also reported a feeling of instability in this trial according to his subjective feedback.

## Discussion

To identify prosthesis control mode switch timings that allow users to safely and seamlessly transition between locomotion tasks, we systematically investigated the effects of different mode switch timings on the users’ walking stability in the task transition periods. The results in Table 1 clearly showed that there was a time window, consisting of 4–5 gait phases, in which switching prosthesis control modes during the transition period did not disrupt the subject’s walking balance in task transitions. This observation was different from the previous assumption that prosthesis control modes must be changed at a specific time to ensure successful and smooth task transitions in ambulation. In addition, our previously defined critical timing was found to be within the safe time window identified in this study. For the transitions RA/RD→W, the latest “safe” timing for switching modes within the studied timings was during foot contact (IDS_2 in Fig 2) in the second gait cycle in the task transition period. This timing was the same as our previously defined “critical timing”, which further validated our previous definition. However, for the transitions W→RA/RD, the change of control mode after the previously defined critical timing (SWF_1) can still allow seamless task transitions. This result implies that this previously defined “critical” timing might not be confined to a small time window. Switching the control mode with a small delay may not affect the user’s performance in task transitions. The observations in this study might be applied to further improve the performance and robustness of hierarchical control of powered lower limb prostheses. For example, the newly updated critical timings can be used to evaluate the response time of different user intent recognition methods in the high-level control for predicting the user’s task transitions [10]. Another example is that for a certain type of task transition, the intrinsic finite-state-machine can prohibit the corresponding mode switch in the gait phases in which such a mode switch disrupts the user’s performance and balance. This approach can eliminate unstable mode switching led by early prediction of task transitions and false alarms occurring in those gait phases.

The difference between mode switch timings that were reported to disturb the subject’s walking balance and ones that allowed safe and seamless task transitions were investigated by examining the mechanical work change at the prosthesis knee joint. From Tables 1 and 2, we observed that for each mode switch timing that caused gait instability, there was at least one gait phase in which the mechanical work change exceeded the tolerable range reported in our previous study [8]. In our previous work, we identified a tolerable range of mechanical work change for each gait phase and observed that large mechanical work changes outside of this range can disrupt the prosthesis user’s gait stability during steady state ambulation. Therefore, we expect that the disruptive effects of “unsafe” mode switch timings on the prosthesis user’s stability during task transitions were also related to the mechanical work change. Our previous work’s findings may also explain the effects of mode switch timing on prosthesis users during task transition periods. This is an important finding because mechanical work change could be a generalized criterion to determine the prosthesis mode switch timings that allow safe and seamless task transitions without extensive testing on human subjects. Comparing the resulting mechanical work change to the suggested tolerable range can verify whether or not a chosen timing or gait phase is safe for prosthesis mode switching. If a large amount of mechanical work change is generated outside of the tolerable range during the selected timing or gait phase, the prosthesis should be prohibited from switching control modes during that time. Furthermore, this criterion is also transferable to other types of powered knee prosthesis designs, control systems, and other types of task transitions.

One limitation of this present study is that the task transitions between walking on level ground and stairs were not considered. This was mainly due to the structural restriction on the current powered knee prosthesis prototype, which could not provide prosthesis users sufficient knee flexion to naturally perform stair-climbing tasks. However, questions about prosthesis control mode switch timing for safe and seamless transitions between level-ground walking and stair ascent/descent could be partially answered based on our previous knowledge and this current study. Our previous study [10] defined critical timings for task transitions between level-ground walking (W) and stair ascent/descent (SA/SD). For example, in the transition W→SA/SD, the critical timing was defined as the beginning of swing phase, which was the timing SWF_1 in this study. To examine whether switching the control mode from W to SA/SD before the defined critical timing could allow safe transition, the generalized criterion (i.e. the mechanical work change) should be used. By measuring the joint torque and joint angular velocity, the mechanical work change during the shifted phases can be estimated. Then, the estimated mechanical work change will be compared with the tolerable range suggested in our previous work [8] to determine whether the mode switch timing is safe. For the transition SA/SD→W, the critical timing was selected at the initial contact of the prosthetic foot on level ground, which was same as the timing IDS_2 in this study. Similarly to W→SA/SD as discussed above, whether or not the control mode can be switched from SA/SD to W after the critical timing can also be determined by investigating the resulting mechanical work change. It is noteworthy that only “half” of the questions could be answered by studying mechanical work change. The answers to the other “half” (switching mode after the critical timing for W→SA/SD and switching mode before the critical timing for SA/SD→W) are still unknown. It is because at these timings, the prosthesis user is still negotiating with the stairs. The factors that determine successful and safe stair transitions not only depend on the prosthesis control, but also relate to the complex external environment (such as stair step height) and the prosthesis user’s status (e.g. hip flexion angle). Although a recent study [55] observed that delayed mode switching during the transitions to stair walking substantially disturbed the prosthesis user’s balance and recovery, no systematic investigation was conducted to determine how much delay can be disruptive for the task transitions. Therefore, appropriate mode switch timing during stair transitions requires further investigation.

To study the effects of different mode switch timings on prosthesis users, we evaluated users’ ability to successfully complete the task transitions and gait stability during transition periods. The results demonstrated that switching control modes either too early or too late disturbed user’s walking stability; however, none of the tested timings terminated the continuous task transitions. This indicated that although switching the prosthesis mode at inappropriate timings disturbed the subjects’ gait stability, they were still able to recover from the disturbances and complete the transitions between walking on level-ground and ramps without stopping. Since walking on stairs is more demanding locomotor task than walking on inclines, switching prosthesis modes too early or too late could cause the user to trip on stairs and eventually fail to complete the task transitions. Therefore, we expect that the success rate of performing seamless task transitions could be a more meaningful metric for evaluating a user’s performance of level-ground walking and stair climbing transitions.

When comparing the gait stability evaluation results obtained from the subjective feedback and the objective quantification, we found that all the mode switch timings that disturbed the subjects’ walking balance in terms of objective quantification index (i.e. full-body angular momentum) were also reported as unstable according to the subjective feedback from the subjects; however, not all of the studied timings that caused subjective feeling of instability were observed to disturb the subject’s full-body angular momentum. This may imply that the subjective feeling from the subjects was a more sensitive measure of walking balance than the detection threshold of the objective quantified balance index used in this study. We thought the subjective feedback was more clinically relevant for evaluating a user’s gait stability. While some mode switch timings did not obviously disturb subjects’ whole-body angular momentum, these mode switch timings may still have elicited insecure feelings of lost walking balance in the subjects, potentially lowering their confidence to use the powered prosthesis to transition between different tasks.

This study attempted to address a critical issue related to the control of powered knee prostheses: the timing when switching prosthesis modes can allow safe and seamless task transitions. Transitions from one task to another are frequently encountered and especially challenging for lower limb amputees during daily ambulation. It has been reported that falls most commonly occur during the first three steps after changing terrains [25, 26], mainly due to high biomechanical demands and poor task planning. Hence, ensuring the prosthesis user’s safety and reducing the risk of falling during task transitions is one key point in the design of powered prosthetic legs. By triggering prostheses to switch modes at appropriate times, prosthesis users will be able to safely and seamlessly transition between different tasks and therefore greatly enhance their confidence in using prosthetic devices.

To summarize, this study identified a time window in which switching the powered transfemoral prosthesis control mode allowed the prosthesis users to change between different locomotion tasks safely and seamlessly. The results of this study could provide an important guidance and criterion for powered transfemoral prostheses to determine the mode switch timing, and further enable the prosthesis users to safely and seamlessly transition between different tasks. Our future work will focus on testing more types of task transitions (such as transitions between level-ground walking and climbing stairs), developing and evaluating hierarchical prosthesis control, incorporating the results of this study, and improving the control robustness to deal with high-level control errors and delays.

## Conclusion

The effects of control mode switch timing on the prosthesis and the user’s walking performance and balance during task transitions were systematically investigated in this study. Five AB subjects and two TF amputees were recruited and tested while wearing a powered knee prosthesis prototype. The prosthesis control mode was switched by a locomotion mode simulator at predefined timings when the subjects performed four types of ambulatory task transitions. The mechanical work generated at the prosthetic knee and the user’s performance and balance in task transitions were quantified. The results showed that a time window existed within which switching the prosthesis control mode did not disturb the user’s walking balance in task transitions. In addition, a generalized criterion based on mechanical work change was proposed to determine the appropriate timing for mode switching. This study could provide important guidance for future powered prosthetic leg control, and further aid the future design of prostheses that are functional and safe-to-use.
